# Spatial and epidemiological mapping of tuberculosis on an Amazon island: effect of the COVID-19 pandemic

**DOI:** 10.1590/1414-431X2025e14015

**Published:** 2026-01-30

**Authors:** V.S. Silva, B.V.J. Gomes, B.C. Nascimento, S.R.O. Cardoso, C.R. Mesquita, R.J.P.S. Guimarães

**Affiliations:** 1 Faculdade de Enfermagem, Universidade Federal do Pará, Belém, PA, Brasil; 2 Programa de Epidemiologia e Vigilância em Saúde, Instituto Evandro Chagas, Secretaria de Vigilância em Saúde, Ministério da Saúde, Ananindeua, PA, Brasil

**Keywords:** Tuberculosis, Epidemiological surveillance, COVID-19, Geographic mapping, Communicable diseases

## Abstract

With the emergence and rise of the COVID-19 pandemic in 2020, many health services were interrupted and reallocated due to system overload. Tuberculosis (TB) care was one of the affected services during this period, especially in regions with greater social vulnerabilities. Thus, this study aimed to describe the distribution of TB cases before and during the COVID-19 pandemic on an island in the Brazilian Amazon. This quantitative descriptive retrospective study evaluated the distribution of 797 new confirmed and notified cases of TB in residents of the sixteen municipalities in the region of Marajó Island (Pará, Brazil), from 2017 to 2022. The data were obtained from the Development and Administration Company of the Metropolitan Area of Belém and Google Earth, using the ArcGIS and TerraView software for georeferencing notification points in each municipality of the archipelago. The Kernel density estimator and scan statistics were used to analyze the point patterns. Almost all municipalities in the archipelago showed variations during the study years. The scan statistics showed a greater number of cases in the pre-pandemic years of 2017-2019. These data indicated that the factors related to the increase and decrease in the number of cases must be analyzed, as the decrease may be related to the underreporting of patients due to the lack of access to health resources in more isolated areas.

## Introduction

Tuberculosis (TB) is an infectious disease caused by *Mycobacterium tuberculosis*. Its prevalence is directly associated to social determinants such as high population density and house clustering, which can facilitate the transmission of this airborne disease. Moreover, low economic conditions can hinder diagnosis and adherence to treatment regimens, particularly when compounded by limited access to health services (1).

TB is highly associated to high levels of social inequality, historically affecting groups with lower education levels, lower income levels, and more limited access to health services (2,3). In addition to these important social vulnerabilities, these areas also often lack adequate public policy coverage. As a result, these regions are more likely to experience precarious health conditions, since investments from the Brazilian Unified Health System (SUS) are hindered by both physical adversities such as rivers and forests, as well as the quality of the service offered, transportation, and infrastructure in remote areas (4,5). Therefore, the need for both effective strategies to prevent and combat TB and a national effort to reduce extreme poverty is evident. This includes expanding and strengthening social protection policies and addressing discrimination and racism (6).

In 2018, the United Nations held its first meeting on TB, where discussions focused on advancing social protection and achieving universal coverage to combat the disease until 2030. However, in 2020, the pandemic adversely affected global progress in TB prevention, diagnosis, and treatment. Throughout the pandemic, significant deficits were exposed in the capacity of health systems worldwide to continue their disease control programs, as the majority of health services were focused on combating COVID-19. From this perspective, areas with existing challenges in healthcare coverage (as in the Marajó region) experienced exacerbated difficulties during the pandemic (7,8).

In Pará, 49.4 cases of TB per 100,000 inhabitants were documented in 2022, being among the 13 states with an incidence rate above the national average of 36.3 cases per 100,000 inhabitants. Furthermore, Pará ranks sixth in the number of TB cases, following Amazonas, Roraima, Rio de Janeiro, Pernambuco, and Acre (9).

Pará has 144 municipalities and is currently the 9th most populous state in the country. The state is surrounded by islands with poor infrastructure and socioeconomic conditions. The Marajó Island is located in the “Golfão Marajoara” of the Amazon region, which has 16 municipalities in 40,100 km^2^. The island lacks advanced healthcare services, with 6 of the 16 municipalities having no hospitals and inadequate primary care services. Furthermore, the primary sources of income in the region are fruit extraction and fishing. The region's economy also relies on cattle and buffalo livestock farming and the production of their derivatives (milk, cheese, and leather) (10).

Among the islands of Pará neglected by health policies, Marajó Island is particularly notable for its low levels of development and precarious living conditions. These challenges include difficulties in mobility and access to health services, which hinder the effectiveness of TB control measures (11). The difficulties are exacerbated by socioeconomic disadvantages and limited transportation options due to the presence of several rivers, requiring the use of boats, which are often inadequate and overcrowded (12).

Moreover, the Municipal Human Development Index factor of the archipelago is among the lowest in the state of Pará. The low educational levels in the region affect individuals' understanding of TB diagnoses and their adherence to treatment (11).

On December 31, 2019, the World Health Organization (WHO) was notified about the high incidence of pneumonia in the city of Wuhan, China. After 7 days, a new strain of coronavirus was identified by authorities, named severe acute respiratory syndrome coronavirus 2. On March 11, 2020, the WHO declared COVID-19 a pandemic, recognizing several viral outbreaks in various countries and regions worldwide (13). The COVID-19 pandemic led to significant disruptions in TB services due to healthcare system overload, social distancing measures, and the reallocation of resources to combat the pandemic. As a result, both the availability and demand for TB diagnosis and treatment were adversely affected, potentially contributing to increased transmission, the emergence of drug resistance, and increased disease severity (14).

From this perspective, this study aimed to describe the TB epidemiological and spatial data on Marajó Island in the pre-COVID-19 pandemic years from 2017 to 2019 and during the pandemic from 2020 to 2022.

## Material and Methods

This ecological descriptive retrospective study used a quantitative approach. The study examined the distribution of 797 new confirmed and notified cases of TB among the residents of the sixteen municipalities of the Marajó Island (Pará, Brazil), from 2017 to 2022. The epidemiological data were obtained from the Notification Sheets of the Disease Information and Notification System, provided by the State Department of Public Health of Pará.

The database organization and graphic construction were performed using Microsoft Office Excel Software, 2019. Population data were obtained from the 2010 Demographic Census and the population estimates provided by the Brazilian Institute of Geography and Statistics.

Georeferencing was carried out using the latitude and longitude coordinates of the reported cases, based on data from the Development and Administration Company of the Metropolitan Area of Belém and Google Earth. This process utilized ArcGIS and TerraView software.

The Kernel density estimator (KDE) and scan statistics were used to analyze the point patterns. The KDE used density parameters classified into four categories, each represented by a specific color: low (green), medium (yellow), high (orange), and extremely high (red).

In the scan method, a circle can encompass various sets of neighboring areas to determine whether the incidence rate is higher within the circle compared with that in the region outside it. The analysis was performed using the SatScan software with two adaptive radii: 39.47 and 86.96 km. A Poisson model was used with the significance level set a P value of <0.05.

The incidence coefficient (CD_TB) for each municipality was calculated by dividing the number of TB cases reported in a given year by the municipality's population data from the 2010 Census, then multiplying the result by 100,000. Subsequently, the percentage variation in the incidence rate was calculated by dividing the difference between the average incidence rates in the pandemic and pre-pandemic periods by the average incidence rate in the pre-pandemic period and multiplying the result by 100 (%).

The current study included individuals diagnosed with TB from 2017-2022 residing in any of the 16 municipalities of Marajó Island (Afuá, Anajás, Bagre, Breves, Cachoeira do Arari, Chaves, Curralinho, Gurupá, Melgaço, Muaná, Ponta de Pedras, Portel, Salvaterra, Santa Cruz do Arari, São Sebastião da Boa Vista, and Soure). Residents with incomplete data were excluded.

**Figure 1:**
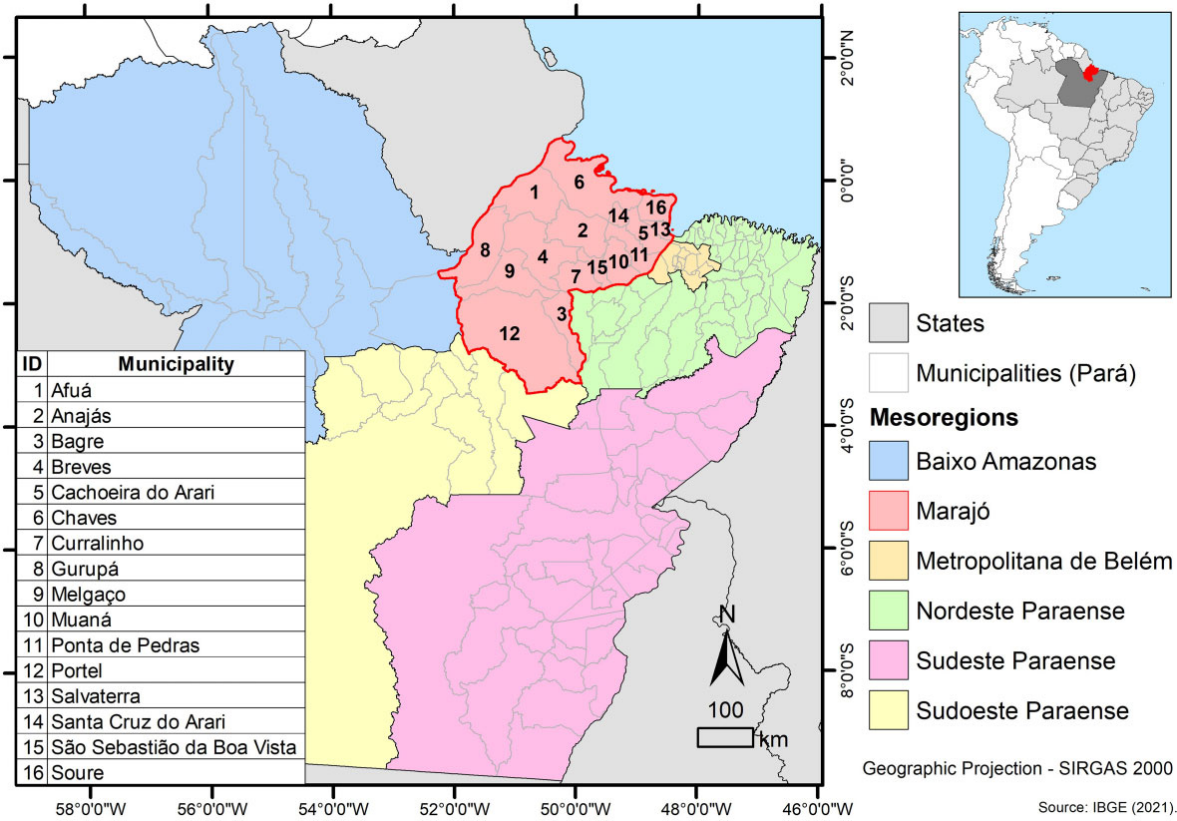
Mesoregion of Ilha do Marajó. Source: Instituto Evandro Chagas (IEC), Geoprocessing Laboratory, 2023.

The Marajó Island (Figure 1) is part of the Marajó mesoregion of the state of Pará. The island complex formed by 16 municipalities covers a total area of 40,100 km^2^ and is divided into three microregions (Figure 1). The Portel microregion has four municipalities (Bagre, Gurupá, Melgaço, and Portel). The Furo de Breves microregion consists of five municipalities (Afuá, Anajás, Breves, Curralinho, and São Sebastião da Boa Vista). Lastly, the Arari microregion comprises seven municipalities (Cachoeira do Arari, Chaves, Muaná, Ponta de Pedra, Salvaterra, Santa Cruz do Arari, and Soure) (15).

The statistical analyses comprised absolute (n) and relative frequencies (%) and assessed the potential growth trends through time. The incidence rate was graphically presented to highlight the differences between the years before (2017, 2018, and 2019) and during the pandemic (2020, 2021, and 2022). For the scan statistics, the clusters were classified by time (cluster 1) and space (cluster 2).

This study met the ethical requirements of the Declaration of Helsinki, the Nuremberg Code, and the norms of Resolutions 466/2012 and 510/2016 of the National Health Council and was approved by the CAAE Ethics and Research Committee (21209919.6.0000.5174).

## Results

**Figure 2:**
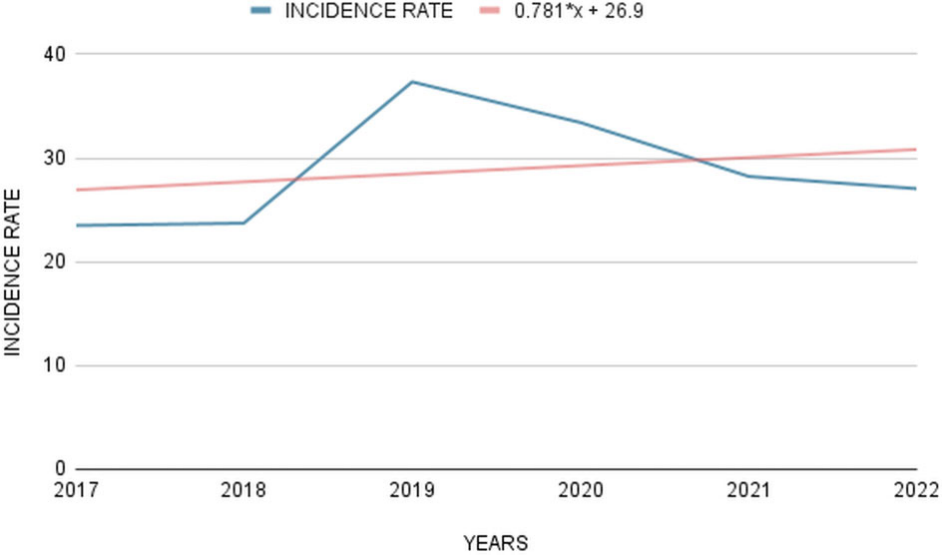
Tuberculosis incidence rates (per 100,000 inhabitants) throughout 2017 to 2022 and the linear regression model (in red). Source: Secretariat of Public Health of Pará, 2022.

TB incidence rate started to increase in 2017 and peaked in 2019 (37.34; 21.55%), before the COVID-19 pandemic. During the pandemic period, TB incidence decreased, dropping to 33.42 (19.29%) in 2020 and continued to decrease until 2022 (Figure 2). Despite this observed decrease, the overall trend line across all the years shows an upward statistical projection, indicating a forecasted increase in TB cases in the coming years and this may be related to factors that impacted TB detections and notifications during the pandemic period, such as discontinuation of treatments or even patients who recovered from COVID-19 and became infected with TB. Therefore, proactive measures must be taken to monitor and control TB, aiming to prevent an increase in cases in future years.

Among the sixteen municipalities in Marajó Island, Bagre had the highest incidence rate before the pandemic period (72.63; 16.1%). During the pandemic, the municipality of Santa Cruz do Arari reported the highest TB incidence rate (57.23; 12.1%). Notably, the municipality of Chaves had minimal variation in incidence rates, with changes occurring only after the onset of the COVID-19 pandemic (1.59; 0.34%) (Table 1).

**Table 1:** Tuberculosis incidence rates in the pre- and post-pandemic periods in Marajó Island, PA.

Municipalities of residence	Incidence rate* (Pre-pandemic)	Incidence rate* (Pandemic)	Variation (%)
Afuá	13.32	23.78	78.53
Anajás	37.70	21.54	−42.86
Bagre	72.63	25.14	−65.39
Breves	32.31	23.69	−26.68
Cachoeira Do Arari	19.57	30.98	58.30
Chaves	0.00	1.59	0.00
Curralinho	14.01	51.37	266.67
Gurupá	29.82	30.97	3.86
Melgaço	12.09	24.19	100.08
Muaná	28.26	27.29	−3.43
Ponta De Pedras	29.49	19.23	−34.79
Portel	11.50	27.47	138.87
Salvaterra	44.59	33.03	−25.93
Santa Cruz Do Arari	49.05	57.23	16.68
São Sebastião Da Boa Vista	27.65	33.47	21.05
Soure	28.98	42.03	45.03

*Per 100,000 inhabitants. Source: Secretariat of Public Health of Pará, 2022.

Almost all study municipalities exhibited variations in TB detection rate. Nine municipalities had an increase in the TB detection rate, ranging from 3.86 in Gurupá to 226.67 in Curralinho. Only six municipalities showed a decrease, ranging from 3.43 in Muaná to 65.39 in Bagre. Chaves was the only municipality with no variations from 2017 to 2022.

Kernel analysis indicated that the most significant variations occurred between 2017 and 2018, during which Muaná, Ponta de Pedras, and São Sebastião da Boa Vista had a decrease in cases, which then increased in 2020 and 2022.

**Figure 3:**
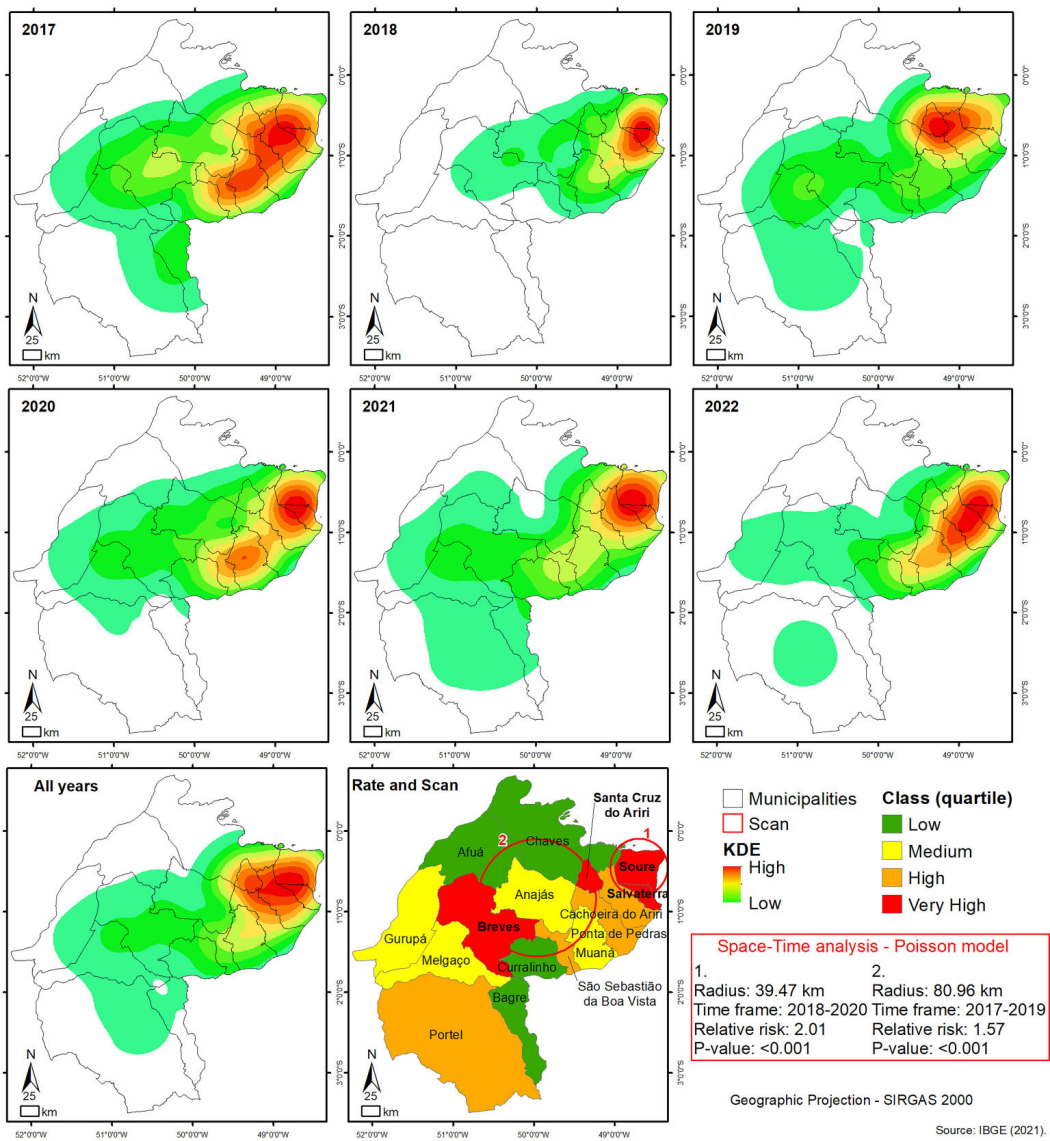
Choropleth maps of tuberculosis incidence rates in Marajó Island, 2017-2022. Source: Secretariat of Public Health of Pará, 2023.

Most of the cases occurred in Soure, Salvaterra, Cachoeira do Arari, Ponta de Pedras, and Muaná, which are adjacent to the metropolitan region of Belém. This proximity likely facilitates TB case notifications on the Island, assuming that the population in these regions seeks more qualified treatment for TB in the capital. However, the Kernel analysis revealed that municipalities bordering other states (Afuá and Chaves) have minimal TB detection rates. It is believed that the residents in these municipalities seek treatment solutions in regions closest to their locations and realities (Figure 3).

The scan statistics identified the clusters with the highest number of cases. The first cluster, with a radius of 39.47 km, was on the border of Soure and Salvaterra and was observed from 2018 to 2020. The second cluster, with a radius of 80.96 km, occurred from 2017 to 2019 and covered Anajás, parts of Breves, Chaves, Afuá, Curralinho, Cachoeira do Arari, Ponta de Pedras, and São Sebastião da Boa Vista.

Furthermore, the Poisson model was employed to estimate the probability of TB occurrence and assess the relative risk of contamination of regions within radii 1 and 2 compared with other areas. Ray 1 had a risk of 2.01, while ray 2 had a risk of 1.57, with both results showing statistical significance (P<0.001).

## Discussion

The results showed that the highest incidence rate was recorded in 2019, followed by a decline beginning in 2020 during the pandemic. Additionally, nearly all sixteen municipalities in the archipelago experienced variations in TB incidence throughout the study period. This research provides valuable insights by reflecting on the impact of COVID-19 on TB epidemiology in a neglected region of the Amazon. The study findings serve as a basis for developing health strategies to control TB rates in these areas.

Socioeconomic factors are key social determinants influencing the spatial distribution of neglected tropical diseases, such as TB. Poor living conditions, including inadequate infrastructure, basic sanitation, and education, hinder individuals' understanding of disease prevention measures (16). For example, the municipality of Santa Cruz do Arari, which had the second-highest incidence of TB before and after the pandemic, is notably disadvantaged: approximately 60.5% of its population earns less than half the minimum wage per capita. Hence, the municipality was classified as one of the poorest in the Marajó region, with an Human Development Index of 0.557, according to the last census (15).

With regard to TB incidence during the pandemic, the number of notifications declined in 2020. This decline may be attributed to the significant changes in health services during the peak of the pandemic. Studies suggest that healthcare facilities redirected resources to combat COVID-19, causing a considerable impact on TB screening, diagnosis, treatment, and prevention services. This shift likely contributed to the underreporting of cases (17).

In addition to the lack of TB confirmatory tests, the clinical similarities between TB and COVID-19 contributed to reporting errors. Differentiating between TB and COVID-19 was challenging due to their overlapping clinical symptoms, such as cough, fever, and dyspnea. These similarities often confused professionals, complicating diagnosis. As a result, the risk of administering inappropriate treatments increased, as the true nature of the patient's condition might not have been accurately identified (18).

To contain the spread of the new coronavirus, the Brazilian government advised the population to stay at home. However, many informal workers in Brazil could not follow this recommendation, highlighting the increased social vulnerability during the COVID-19 period (19). Studies suggest that although the Brazilian confinement measure, also adopted in other countries, effectively reduced the number of COVID-19 cases, important indicators of tuberculosis were drastically affected during the pandemic (20,21).

The underreporting of TB cases also resulted from a reduced demand for care, as confinement measures were implemented to prevent overcrowding health centers (22). Of the critical periods, 2020 was notable for the emergence of the pandemic, a substantial increase in the number of cases, and the first emergency declaration in the country; these events led to a significant reduction in individuals seeking health services (23).

The observed decrease in detection rates during the COVID-19 pandemic may have resulted in the higher number of cases in subsequent years, as untreated and unidentified individuals with pulmonary or laryngeal TB continued to spread the disease. This scenario can be related to the growing trend observed in this research, which projects an increase in TB cases in the coming years. In addition, a study points to an increase in TB infection among individuals who have recovered from COVID-19, reinforcing the trend. One of the points highlighted was the corticosteroid treatment for COVID-19, which can cause immunosuppression and is associated with a greater susceptibility to acquiring TB (24).

Several factors have influenced and contributed to the increase in TB cases after COVID-19. During this period, there was a significant reduction in the performance of MDR-TB tests, resulting in late diagnoses and, consequently, delayed treatment initiation. Thus, undiagnosed individuals tended to present more severe symptoms, due to the lack of adequate treatment for TB. The identification and reporting of these cases could have occurred at more advanced stages (7).

The predominance of cases reported in municipalities near Belém is due to the easier access to healthcare services in the capital. Although geographically close to the study region, the trip to Belém often requires lengthy boat journeys, which can facilitate disease transmission. Among the municipalities on Marajó Island, a noticeable flow of people towards Breves reflects the disparities in the quality of health services, with patients travelling between municipalities to access better services (11). The population of municipalities near the border with other states may seek treatment in these states, where the cases are reported, making the analysis of cases impossible.

The reason for the decline in TB on Marajó Island should be examined. The decrease may be associated with underreporting due to the difficulties imposed by the pandemic, such as the lack of access to health resources in isolated areas. Spatial analysis is fundamental to improving the effectiveness of government decision-making, using geographic visualization tools to identify patterns and distributions. This approach helps in urban planning, natural resource management, and the evaluation of public policies. By providing a more detailed view of regional needs and vulnerabilities, spatial analysis helps in predicting risks and implementing preventive measures, which promote more informed, efficient, and responsive governance.

In conclusion, the differences in TB incidence rates are related to the specific characteristics of each municipality, such as economic and spatial factors. Therefore, controlling TB is crucial, as the disease disproportionately impacts impoverished communities, exacerbates poor living conditions, and affects the overall economy.

## Data Availability

All data generated or analyzed during this study are included in this published article.
